# Quality of life and its associated factors among women diagnosed with pelvic organ prolapse in Gynecology outpatient department Southern Nations, Nationalities, and Peoples region public referral hospitals, Ethiopia

**DOI:** 10.1186/s12905-023-02507-9

**Published:** 2023-06-28

**Authors:** Zewdu Tefera, Belsity Temesgen, Mikyas Arega, Tmesgen Getaneh, Aynalem Belay

**Affiliations:** 1 Department of Midwifery, College of Health Science, Dilla University, Dilla, Ethiopia; 2 Department of Midwifery, College of Health Science, Debre Markos University, Debre Markos, Ethiopia; 3 Department of Midwifery, College of Health Science, Wolkite University, Wolkite, Ethiopia

**Keywords:** Pelvic organ prolapse, Quality of life, Southern Ethiopia 2022

## Abstract

**Background:**

Pelvic organ prolapse is an important public health issue that influences millions of women’s lives; through limitations on physical, social, and sexual activities as well as psychological distress. However, there were no reports on the quality of life for women with pelvic organ prolapse in Ethiopia. Therefore this study amid to assess the magnitude of quality of life and its associated factors among women who diagnosed with pelvic organ prolapse in Gynecology outpatient departments in Southern Nations, Nationalities, and Peoples region public referral hospitals; Ethiopia.

**Method:**

An institutional-based cross-sectional study was conducted in Southern Nations, Nationalities, and Peoples region of public referral hospitals from May 1-July 4, 2022 among 419 diagnosed women with pelvic organ prolapse. A validated tool was used to collect the data. The collected data were entered into Epidata version 3.1 and analyzed using the Statistical Package for Social Sciences. Bivariable and multivariable logistic regression was computed. The p-value of < 0.05 was used to declare the final statistical significance.

**Result:**

A total of 409 women with pelvic organ prolapse were included in the study, giving a response rate of 97.6%. The overall poor quality of life was 57.5%. Regarding the quality of life domains; personal relationships (73.6%), were highly affected, and sleep/energy (24.2%) was the least affected domain. Stage III/IV prolapse (AOR = 2.52, 95% CI: 1.34, 4.74), menopause (AOR = 3.21, 95% CI 1.75, 5.97), unmarried women (widowed, divorced) (AOR = 2.81, 95% CI: 1.48, 5.32), and longer duration of prolapse (AOR = 5.8, 95% CI: 3.13, 10.81), were significantly associated with poor quality of life.

**Conclusion:**

More than half of women with pelvic organ prolapse had a poor quality of life. Stage III/IV prolapse, longer duration of prolapse, menopause women, and unmarried women are statistically significant factors for the quality of life of women with Pelvic organ prolapse.

**Supplementary Information:**

The online version contains supplementary material available at 10.1186/s12905-023-02507-9.

## Background

Pelvic organ prolapse (POP) is the descent of female pelvic organs into or through the vagina, including the bladder, uterus, and rectum [1]. This case results from the defect of the pelvic floor support, caused by many risk factors such as vaginal birth, advancing age, and increasing body mass index; atrophic changes caused by aging or estrogen loss, chronic straining, and abnormalities of connective tissue [2, 3].

The affected women may present with signs and symptoms of urinary or fecal loss or retention, vaginal pressure or heaviness, abdominal, low back, vaginal, or perennial pain or discomfort, a mass sensation, difficulty walking, lifting, sitting, and stress or fear related to anxiety about the problem [1, 3, 4]. It is the leading indication for hysterectomy in postmenopausal women and accounts for 15–18% of procedures in all age groups [5]. In the Southern Nation Nationality and Peoples Region of Ethiopia, there were high burdens of pelvic organ prolapse, and the most common cause was old age, long hours of carrying heavy objects, a high parity, a history of home delivery, a history of chronic constipation, and a history of chronic cough [6, 7].

Pelvic organ prolapse is a significant public health issue that affects the lives of millions of women [8]. On average, POP influenced 19.7% of women in developing countries [9]. Similarly in Ethiopia, it influenced 23.52% of women [10]. It has the potential to severely influence women’s health-related quality of life through restrictions on physical, social, and sexual activities, psychological distress, and increased financial burden related to healthcare [11]. In addition, access to health care to manage these conditions is often limited, and women usually have to live with the consequences for the rest of their lives [8].

The quality of life of women with POP varies from country to country based on economic level, lifestyle, educational level, and culture [12]. However, a study showed that globally, the most common predominant risk factor for worsening QoL in women with POP is symptoms of POP [13, 14]. Overall, pelvic floor disorders harm women’s lives, emotions, and quality of life, and they may be associated with a variety of systemic symptoms such as urinary, bowel, and sexual symptoms, which may significantly confront the quality of life of the women [15, 16].

Pelvic organ prolapse highly affects women’s quality of life in developing countries compared to developed countries [17, 18]. However, in developing countries, the degree and consequences of the burden of the disease due to pelvic floor dysfunction, especially on the quality of life, are more poorly understood [19]. In addition, the QoL of POP on women’s health has not yet been recognized as a public health problem in many developing countries. This is because only a few studies have examined POP about the normal deterioration of general health-related QOL in the general population [9, 20].

In developed countries, the quality of life and its associated factor in women with POP is assessed with P-QoL tool and used as a baseline strategy for the treatment of POP, but in developing countries, especially in Africa, including Ethiopia not applicable, this is due to limited information on the quality of life of pelvic organ prolapse [21]. Despite the quality of life allowing the quantification of morbidity, treatment efficacy and also acting as a measure of how lives are affected, and coping strategies, research has usually concentrated on the prevalence, etiology, diagnosis, and management of pelvic floor dysfunction, with limited work being performed on the effects of chronic conditions or their treatment on QoL [22].

Pelvic organ prolapse is one of the sources of severe morbidity and psychological upheaval for the patient, who is often socially withdrawn and stigmatized and negatively influences the socioeconomic and reproductive activity of affected women [23, 24]. However, in Ethiopia, no reports were showing the quality of life of women with pelvic organ prolapse. Measuring the quality of life of women with POP is an important input for policymakers and program implementers to develop strategies.

## Methods

### Study area and period

The study was conducted in South Nations, Nationalities, and Peoples Region (SNNPR). The SNNPR is bordered by Kenya to the south, South Sudan to the west, Gambela regional to the northwest, Oromia region to the north and east, South West region to the Southwest, and Sidama region to the east [25]. The region has 3-referral public hospitals(Dilla university referall, Wolaita Sodo university, Wolkite university) 2 specialized and comprehensive hospitals, 3 general hospitals, 44 primary hospitals, 474 health centers, and 2633 health posts [26]. The previous two-month report in the three referral public hospitals showed that there were four hundred seventy POP cases [27]. The data were collected from May 1, 2022-July 4, 2022 in public referral hospitals, SNNPR; Ethiopia 2022.

### Study population and eligibility criteria

All women who were diagnosed with pelvic organ prolapse in the Gynecology outpatient department of SNNPR public referral hospitals during the data collection period. All women diagnosed with pelvic organ prolapse and who had not gotten treatment before in the Gynecology outpatient department in all selected public referral hospitals were included.

### Sample size and sampling procedure

The sample size was calculated using a single population formula (*n* = (Z α/2)^2^_*_ P (1-P) ⁄ d^2^).

Based on a study conducted in Uganda, the proportion of poor QoL among POP women was 45.5% [28]. Then, using the following assumptions: 95% of a confidence interval, α = 0.05, and margin of error = 5% the sample size required for this study was calculated.

The calculated sample size was 381. By adding 10% of the non-response rate the final sample size was 419.

All three public referral hospitals found in SNNPR were included in this study. The previous two months’ average POP report of each hospital was used to proportional allocate the calculated sample size [27]. Finally, study respondents were selected using a consecutive sampling technique.

### Operational definitions

#### Quality of Life (QoL)

WHO defines the quality of Life as an individual’s perception of their position in life in the context of the culture and value systems in which they live and about their goals, expectations, standards, and concern. It is a multi-dimensional concept that includes domains related to physical, mental, emotional, level of independence, and social functioning [29].

#### Poor quality of life

Greater or equal to the median score of the overall (nine) QoL domains [30, 31].

#### Duration of prolapse: 

The number of months or years from the time pelvic organ prolapse first occurred until now [32].

#### Stages of prolapse

Based on Baden–Walker Halfway Scoring System:

- Stage 0: is no prolapse.
  Stage I: is leading part of the prolapse is more than 1 cm above the hymen.
  Stage II: is the leading edge less than or equal to 1 cm above or below the hymen;
  Stage III: is leading edge is more than 1 cm beyond the hymen, but less than or equal to the total vaginal length;
  Stage IV: is complete eversion [1, 2, 33].

### Data collection tool and procedure

The data collection tool was adopted from the University of Gondar validated tool on prolapse quality of life questionnaire (P-QoL) [34]. An interviewer-administered validated Amharic version questionnaire was used to collect the data. The questionnaire includes 20 items in nine different domains (General Health Condition and POP on the overall life has one item, Role limitation, Social Limitation, Sleep/ Energy and physical limitation has two items, personal relationship and emotion has three items, intensity or Severity of Pain have four items).

The Questions are rated on a four-point Likert scale ranging from one to four, indicating better to worse conditions. This scale was converted into zero to hundred scales. Therefore, the total score is a range of 0–100. Each domain score was obtained by adding the scores of the individual items that comprise the domain. A full-scale score was obtained by the addition of nine-domain items. The lowest total score that can be on the scale is zero, and the highest score is 100. The cut-off point was the median of the total score [35]**.**

Three BSc and MSc midwives were recruited as data collectors and supervisors respectively. The training was given to both data collectors and supervisors by the investigator about the objective of the study, data collection tool, procedure, and how to fill out the questionnaires. All women who were diagnosed with POP in the Gynecology outpatient department of SNNPR public referral hospitals were interviewed after assessing eligibility and obtaining informed written consent and their charts were reviewed to assess the stage of the prolapse.

### Data quality assurance

The training was given to data collectors and supervisors. A data collector was supervised throughout the data collection period. Then, the overall process was coordinated and controlled by the investigator. Investigators, supervisors, and data collectors took a discussion meeting after data collection to ensure completeness. Furthermore, the collected data was Checked and coded and entered into the Epi-data computer program version 3.1 to minimize data entry errors.

### Data processing and analysis

The collected data was entered into the EPI data version 3.1 computer program. Then, it was exported to Statistical Package for social science version 25. Descriptive statistics like frequency and summary statistics were employed to describe the characteristics of the study participants. Multicollinearity of the predictor variable was checked by using the variance inflation factor before binary logistic regression was done, and it was < 2 VIF for all independent variables. All explanatory variables in bivariable logistic regression that fulfill the chi-square static assumption were considered for multivariable logistic regression analysis to control for confounding factors. Adjusted Odds Ratio (AOR) with their corresponding 95% Confidence Intervals (CI) and *p*-value less than 0.05 was used to declare the association between dependent and independent variables. Model fitness was checked by the Hosmer–Lemeshow test, it was fitted with a *p*-value of 0.45; finally presented by figure, table, and graph.

## Result

### Socio-demographic characteristics

A total of 409 women were enrolled in the study, giving a 97.6% response rate. The age range of the participants is 36 to 72 years with a median of 54 years. Most of the respondents 220(53.8%) were married. Additionally, regarding educational status, 204 (49.87%) were non-educated (Table 1).

**Table 1:** Socio-demographic characteristics among women diagnosed with POP in Gynecology outpatient department SNNPR public referral hospitals; Ethiopia 2022 (*n* = 409)

Variables		Frequency (%)
Age in years	35–49	103(25.2)
	50–59	187(45.7)
	≥ 60	119(29.1)
Residency	Rural	304(74.3)
	Urban	105(25.7)
Educational status	Non-formal educated	204(49.88)
	Primary	161(39.36)
	Secondary and above	44(10.76)
Marital status	Married	220(53.8)
	Divorced	124(30.3)
	Widowed	65(15.9)
Occupational status	Farmer	219(53.54)
	Housewife	86(21.0)
	Government employ	46(11.24)
	Merchant	32(7.82)
	Private employ	26(6.4)
Transport accessibility	Constantly	157(38.38)
	Never	145(35.46)
	Rarely	107(26.16)

### Gynecologic and obstetrics characteristics

For most respondents, 251(61.36%) prolapses were greater than two years durations. Regarding stages of prolapse, 153(37.4%) of women had stage III prolapse. Moreover, the parity ranged from 4 to 14 with a mean and standard deviation of (8.23 ± 2.23), and 295(72.1%) of women had ≥ 7 number of childbirth (Table 2).

**Table 2:** Gynecologic & obstetrics characteristics among women diagnosed with POP in Gynecology outpatient department SNNPR public referral hospitals, Ethiopia 2022 (*n* = 409)

Variables		Frequency (%)
Stages of prolapse	Stage I	76(18.6)
	Stage II	71(17.4)
	Stage III	153(37.4)
	Stage IV	109(26.6)
Duration of prolapse	1-2 year	158(38.6)
	> 2 year	251(61.4)
Menopausal status	Menopausal	290(70.9)
	Premenopausal	119(29.1)
Parity	4–6	114(27.9)
	≥ 7	295(72.1)
Decubitus ulcers	Yes	62(15.2)
	No	347(84.8)

### The magnitude of quality of life of women with pelvic organ prolapse

**Fig. 1:**
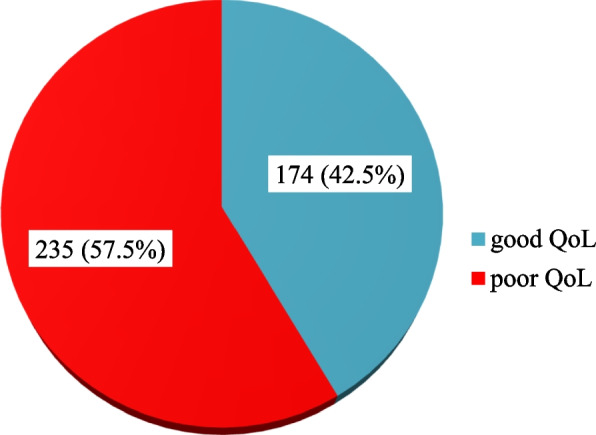
Magnitude of quality of life among women diagnosed with pelvic organ prolapse in Gynecology outpatient department SNNPR public referral hospitals, Ethiopia 2022 (*n* = 409)

According to this study, the magnitude of poor quality of life among women with POP was 235(57.5%) with (95% CI: 52.5, 62.3) (Fig. 1). Personal relationships 301(73.6%), the impact of pop 248(60.6%), and emotional well-being 239(58.4%) were domains of quality of life that had the highest score, whereas social limitation 142(34.7%) and sleep/energy 99(24.2%) had the lowest score (Table 3).

**Table 3:** Quality of life domain score among women diagnosed with pelvic organ prolapse in Gynecology outpatient department SNNPR public referral hospitals, Ethiopia 2022 (*n* = 409)

Domains	QoL domain score	
	Median score	Prevalence QoL domain score above median score (%)
The overall quality of life	66.66	235(57.5)
General health condition	66.66	216(52.8)
Impact of prolapse	66.66	248(60.6)
Role limitation	66.66	233(57.0)
Physical limitation	33.33	187(45.7)
Social limitation	33.33	142(34.7)
Personal relationship	66.66	301(73.6)
Emotion	66.66	239(58.4)
Sleep/energy	33.33	99(24.2)
Severity measure	66.66	238(58.2)

### Factor associated with QoL of women with pelvic organ prolapse

To assess the relationship between various socio-demographic, obstetric, and POP-related factors and quality of life, binary logistic regression was used. The variables that full fill the chi-square static assumption were maternal educational status, residency, marital status, parity, transportation access, menopausal status, duration POP, and stage of POP. Then, using the backward likelihood ratio approach, these variables were subjected to multivariable logistic regression analysis. In the final model, only five variables were present. Model fitness was tested with the Hosmer and Lemeshow Goodness of fit test and fit with a *p*-value of 0.45. Additionally, all independent variables had no multicollinearity with a variance inflation factor value (VIF) < 2.

This study shows that women with stage III/IV prolapse were 2.52 times more likely to have a poor quality of life than those with stage I or II prolapse (AOR = 2.52, 95% CI: 1.34, 4.74). In addition, women in the menopause period were 3.21 times more likely to have a poor quality of life than in the pre-menopause period (AOR = 3.21, 95% CI 1.75, 5.97). Furthermore, the odds of having a poor quality of life were 2.81 times greater for women who were not married (widowed or divorced) than for those who were married (AOR = 2.81, 95% CI:1.48, 5.32). Moreover, women with a longer duration of POP were 5.8 times more likely to have a poor quality of life than their counterparts (AOR = 5.8, 95% CI: 3.13, 10.81) (Table 4).

**Table 4:** Factor associated with QoL among women diagnosed with POP in Gynecology outpatient department SNNPR public referral hospitals, Ethiopia 2022 (*n* = 409)

Variables		Quality of life			
		Frequency (%)		COR(95%CI)	AOR(95%CI)
		Poor	Good		
Menopausal status	Menopause Pre-menopause	197(48.16) 38(9.3)	93(22.74) 81(19.80)	4.51(2.85, 7.13) 1	3.21(1.75, 5.97) 1
Stages of pop	Stage III/IV Stage I/II	184(44.98) 51(12.46)	76(18.59) 98(23.97)	4.65(3.02, 7.16) 1	2.52(1.34, 4.74) 1
Marital status	unmarried Married	145(35.45) 90(22.00)	44(10.76) 130(31.79)	4.76(3.092, 7.32) 1	2.81(1.48, 5.32) 1
Duration of pop	> 2 year ≤ 2 year	200(48.9) 35(8.56)	51(12.47) 123(30.07)	13.78(8.48,22.39) 1	5.81(3.13, 10.81) 1
Residency	Rural Urban	203(49.63) 32(7.82)	101(24.70) 73(17.85)	4.58(2.83, 7.40) 1	2.48(1.158,5.31) 1
Parity	≥ 7 4–6	195(47.68) 40(9.78)	100(24.45) 74(18.09)	3.6(2.29, 5.68) 1	2.04(0.97, 4.32) 1
Transport accessibility	Never Rarely Constantly	110(26.9) 48(11.73) 77(18.83)	35(8.56) 59(14.42) 80(19.56)	3.26(1.99, 5.34) 0.84(0.51, 1.38) 1	1.42(0.81, 4.57) 0.65(0.23, 1.53) 1
Educational status	Non-educated Primary Secondary and above	148(36.20) 68(16.62) 19(4.64)	56(13.70) 93(22.73) 25(6.11)	3.47(1.77, 6.80) 0.96(0.49, 1.88) 1	0.54(0.19, 1.51) 0.607(0.21, 1.68) 1

## Discussion

This study showed that the overall poor quality of life among women who had pelvic organ prolapse was 57.5% (95%CI: 52.5, 62.3). This indicated that, although pelvic organ prolapse is a benign case, it highly influences the quality of life of women. This finding is in line with a study conducted in France 54.5% [36]; Pakistan 60.8% [37], and Slovakia 52.8% [12]. This is due to the fact that, although the quality of life of women with POP varies from country to country based on economic level, lifestyle, educational level, and culture, the most common predominant risk factors for worsening QoL in women are pelvic organ prolapse symptoms [13, 14].

On the other side, it is higher than a study conducted in Uganda 45.5% [28], and Ghana 39.4% [38]. This difference is due to QoL domain and item differences and sample size differences.

In addition, this study showed that the poor quality of life of women with POP was significantly associated with advanced stages of prolapse. This finding is supported by studies conducted in South Africa [30], Taiwan [39], Thailand [16], Nepal [40], the USA [41], Italy [42], and London [31].

This is due to the fact that as the prolapse advanced, secondary consequences, including decubitus ulcers, bowel symptoms, urinary symptoms, abdominal symptoms, and vaginal symptoms, increased, which worsened the quality of life [31, 38, 40].

Moreover, this study showed that there was a significant association between the poor quality of life of women with POP and longer durations of prolapse. This finding is supported by studies conducted in Pakistan [37], France [13], and Bangladesh [43]. This is because, as the duration of the disease increased, there was progressive disability. In addition, as the duration of the prolapse increased, there were advanced stages of prolapse that caused a symptomatic prolapse that worsened women’s health conditions [44].

Furthermore, this study showed that women’s being unmarried was strongly associated with poor quality of life. This finding is in line with studies conducted in Bangladesh [43]. In fact being in a relationship, could be considered a buffer mechanism against psychological illnesses, reducing the likelihood of developing depressive symptoms, and isolation [30]. In addition, lonely women with pelvic organ prolapse had double responsibility to lead their lives; this condition could be worse for their lives.

Moreover, this study showed that menopausal women were significantly associated with poor quality of life. This finding is supported by studies conducted in Turkey [20]. This is due to the fact that as the women entered the menopausal stage, there were physiological changes resulting in menopausal symptoms and worsening of the prolapse, which is a double burden that worsens the quality of life [1].

## Conclusion

According to this study, more than half of women with pelvic organ prolapse had a poor quality of life. Stage III/IV prolapse, longer duration of prolapse, menopausal period, and unmarried women (widowed, divorced) were significantly associated with poor quality of life.

### Recommendation

To the South Nation Nationalities and Peoples of Ethiopia region health bureau:

- Offering the possibility of early diagnosis and treatment for women with POP that has been hidden in the community

To researcher:

- Researches need on why women with pelvic organ prolapse are not early attending health care facilities (Time to health-seeking behavior among women with POP and its factors).
- Further community based study

## Supplementary Information

**Additional file 1.**

## Data Availability

The datasets analyzed during the current study are available from the corresponding author upon reasonable request.

## Abbreviations

AOR: Adjusted Odds Ratio,
HRQoL: Health related quality of life
OPD: Outpatient Department
POP: Pelvic Organ Prolapse
QoL: Quality of Life
SNNPR: Southern Nations, Nationalities, and Peoples Region
