# Supplementary material for: Quality of life and its associated factors among women diagnosed with pelvic organ prolapse in Gynecology outpatient department Southern Nations, Nationalities, and Peoples region public referral hospitals, Ethiopia
Source: BMC Womens Health. 2023 Jun 28;23:342. doi: 10.1186/s12905-023-02507-9 (PMC10308721; doi:10.1186/s12905-023-02507-9)
Supplement: Supplementary file 1 — Additional file 1. [file 12905_2023_2507_MOESM1_ESM.docx]

# APPENDIXES

**Annex I Information sheet and consent form**

**Introduction:** Hello, how are you? My name is ___________ I am working as a data collector in a survey conducted by Zewdu Tefera at referral public hospitals in SNNPR. The research supported in collaboration of Debre Markos University, College of Health Sciences, Midwifery Department. You are invited to participate in this study and I kindly request your active involvement in this survey to provide me with the necessary information. So thank you for your contribution

**Study topic:** Quality of life and its associated factors among women diagnosed with pelvic organ prolapse in public referral hospitals, SNNPR, Ethiopia 2022

**The objective of the study:** To assess the quality of life and its associated factors among women diagnosed with pelvic organ prolapse in public referral hospitals, SNNPR, Ethiopia 2022.

**Study period**: May 1- July 4, 2022

**Process of study**: I am going to ask you some questions that are not difficult to answer. You do not have to answer any question that you do not want to answer and you may end this interview at any time you want to. I would appreciate your help in responding to this survey question. This interview may take 15-20 minutes.

**Benefit and risk of the study**: There is no payment or any special privilege given to you, but your honest answers to these questions will help the study to identify the magnitude and impact of pelvic organ prolapse on women's health and the importance for management strategy. If you are not feeling good at any time, please don’t worry to ask to stop the procedure.

**Confidentiality**: Certainly, I assure you that your name or your name will not be mentioned/ recorded anywhere. The confidentiality of the information you provided to me will be maintained and could not be accessed by a third party but it has been used for research only and burnt by the end of the survey. If you have any questions regarding this study, you can call me at **0915717455.** After I get clear information and understanding about this interview: I accept the interview I reject the interview

Data collector name ___________Signature ______Date of data collection_________

Checked by supervisor; Name ________________Signature_________

Questionnaire code________

**Annex II Questionnaire English Version**

The questionnaire used to assess the quality of life and its associated factor among pelvic organ prolapse women in public referral hospitals, SNNPR, Ethiopia 2022

| **Part I: Socio-economic & demographic characteristics** | |  |  | | | | |
| --- | --- | --- | --- | --- | --- | --- | --- |
|  |  |  |  |  | |  |  |
| No | Question |  | Answers |  | |  | Remark |
|  |  |  |  | | |  |  |
| 101 | How old are you? |  | _____completed Years | | |  |  |
|  |  |  |  | | |  |  |
| 102 | What is your religion? |  | 1. Orthodox | | |  |  |
|  |  |  | 1. Muslim | | |  |  |
|  |  |  | 1. Protestant | | |  |  |
|  |  |  | 1. Others(specify) | | |  |  |
|  |  |  |  | | |  |  |
| 103 | Where is your residency |  | 1. Urban 2. Rural | | |  |  |
| 104 | What is your marital status? |  | 1. Single | | |  |  |
|  |  |  | 1. Married | | |  |  |
|  |  |  | 1. Divorced | | |  |  |
|  |  |  | 1. Widowed | | |  |  |
|  |  |  | 1. Others(Specify) | | |  |  |
|  |  |  |  |  | |  |  |
| 105 | What is your educational level? |  | 1. Non-educated 2. No formal education |  | |  |  |
|  |  |  | 1. Primary | | |  |  |
|  |  |  | 1. Secondary | | |  |  |
|  |  |  | 1. Diploma and above | | |  |  |
|  |  |  |  | | |  |  |
|  |  |  |  | | |  |  |
| 106 | What is your occupation? |  | 1. Merchant | |  | | |
|  |  |  | 1. Government employee | |  | | |
|  |  |  | 1. Housewife | |  | | |
|  |  |  | 1. Farmer 2. Private employee | |  | | |
|  |  |  | 1. Others(specify) | |  | | |
|  |  |  |  | |  | | |
|  |  |  |  |  |  | | |
| 107 | What is your estimated monthly income? | | ------------ | |  | | |
| 108 | Have you got transport access as you wish? | | 1. Never 2. Rarely 3. Constantly | |  | | |

**Part II**

**To assess the quality of life with POP-related factor**

| **No** | **Question** | **Questionaries’ code and answer** |  |
| --- | --- | --- | --- |
| 201 | How many months/year is your prolapse? | **-----**completed in year |  |
| 202 | How many times have you given birth? | …………. |  |
| 203 | Have you seen menstruation for the last one year? | 1. Yes 2. No |  |
| 204 | Stages of the prolapse | 1. Stage I 2. Stage II 3. Stage III 4. Stage IV |  |
| 205 | Does the patient have a decubitus ulcer? | 1. Yes 2. No |  |

**Part III**

**To assess the quality of life of pop**

Please answer this questionnaire even if you feel you do not have symptoms of a prolapse

| Sr.no | QoL domain question | | | | | | **Questioner's answer and code** | | | | | | | | | | |  |
| --- | --- | --- | --- | --- | --- | --- | --- | --- | --- | --- | --- | --- | --- | --- | --- | --- | --- | --- |
|  | 1. **General Health Condition** | | | | | | 1 | | | | | | 2 | 3 | | 4 | |  |
|  |  |  |  |  |  |  | **Very good** | | | | | **good** | | **Poor** | | **Very poor** | |  |
| 1.1 | How would you describe your health at present? Please tick one answer | | | | | |  | | | | |  | |  | |  | |  |
|  | 1. POP on the overall of life | | | | | | 1 | | | | | 2 | | 3 | | 4 | |  |
|  |  |  |  |  |  |  | **not at all** | | | | | **slightly** | | **Moderately** | | **A lot** | |  |
| 2.1 | How much do you think your prolapse problem affects your life? Please tick one answer | | | | | |  | | | | |  | |  | |  | |  |
|  |  | 1. Role limitation | | | | | | 1 | | | 2 | | | 3 | | 4 | | |
|  |  |  |  |  |  |  |  | **not at all** | | | **slightly** | | | **moderately** | | **A lot** | | |
| 3.1 | To what extent does your prolapse affect your household task (e.g. cleaning, shopping etc?) | | | | | | | |  | |  | | |  | |  |  |  |
| 3.2 | Does your prolapse affect your job or your  normal daily activities outside the home? | | | | | | | |  | |  | | |  | |  |  |  |
|  | 1. Physical Limitation | | | | | | | | 1 | | 2 | | | 3 | | 4 |  |  |
|  |  |  |  |  |  |  |  |  | **not at all** | | **slightly** | | | **moderately** | | **A lot** |  |  |
| 4.1 | | | Does your prolapse affect your physical  activities (e.g. going to walk, run, sport, gym, etc. | | |  | | | | |  | | |  | |  |  |  |
| 4.2 | | | Does your prolapse affect your ability to travel? | | |  | | | | |  | | |  | |  |  |  |
|  | | | 1. Social Limitation | | | **not at all** | | | | | **slightly** | | | **moderately** | | **A lot** |  |  |
| 5.1 | | | Does your prolapse limit your social life? | | |  | | | | |  | | | |  |  |  |  |
| 5.2 | | | Does your prolapse limit your ability to see /visit friends? | | |  | | | | |  | | | |  |  |  |  |
|  | | | 1. Personal Relationship | | | 1 | | | | | 2 | | | | 3 | 4 |  |  |
|  |  |  |  |  |  | **not at all** | | | | | **slightly** | | | | **moderately** | **A lot** |  |  |
| 6.1 | | | Does your prolapse affect your  relationship with your partner? | | |  | | | | |  | | | |  |  |  |  |
| 6.2 | | | Does your prolapse affect your sex life? | | |  | | | | |  | | | |  |  |  |  |
| 6.3 | | | Does your prolapse affect your family life? | |  | | | | | |  | | | |  |  |  |  |
|  | | | 1. Emotion | 1 | | | | | | | 2 | | | | 3 | 4 |  |  |
|  |  |  |  | **not at all** | | | | | | | **slightly** | | | | **moderately** | **A lot** |  |  |
| 7.1 | | | Does your prolapse make you feel  depressed? | |  | | | | | |  | | | |  |  |  |  |
| 7.2 | | | Does your prolapse make you feel  anxious or nervous? | |  | | | | | |  | | | |  |  |  |  |
| 7.3 | | | Does your prolapse make you feel  bad about yourself? | |  | | | | | |  | | | |  |  |  |  |
|  | | | 1. Sleep/ Energy | | 1 | | | | | 2 | | | | | 3 | 4 |  |  |
|  |  |  |  |  | **not at all** | | | | | **slightly** | | | | | **moderately** | **A lot** |  |  |
| 8.1 | | | Does your prolapse affect your sleep? | |  | | | | | |  | | | |  |  |  |  |
| 8.2 | | | Do you feel worn out /tired? | |  | | | | | |  | | | |  |  |  |  |
|  | | | 1. Intensity or Severity of Pain | | 1 | | | | | | 2 | | | | 3 | 4 |  |  |
|  |  |  |  |  | **Never** | | | | | | **sometimes** | | | | **often** | **All the time** |  |  |
| 9.1 | | | Use tampons/pads/firm knickers to help. | |  | | | | | |  | | | |  |  |  |  |
| 9.2 | | | Do you push up the prolapse? | |  | | | | | |  | | | |  |  |  |  |
| 9.3 | | | Pain /discomfort due to the prolapse? | |  | | | | | |  | | | |  |  |  |  |
| 9.4 | | | Does the prolapse prevent you from standing? | |  | | | | | |  | | | |  |  |  |  |

Thank you!!
